# Expansion and activation of CD4^+^CD25^+^ regulatory T cells in *Heligmosomoides polygyrus* infection

**DOI:** 10.1002/eji.200636751

**Published:** 2007-07

**Authors:** Constance A M Finney, Matthew D Taylor, Mark S Wilson, Rick M Maizels

**Affiliations:** Institute of Immunology and Infection Research, University of EdinburghEdinburgh, UK

**Keywords:** Mesenteric lymph node, Parasitic helminth, T cells

## Abstract

Regulatory T cell responses to infectious organisms influence not only immunity and immunopathology, but also responses to bystander antigens. Mice infected with the gastrointestinal nematode parasite *Heligmosomoides polygyrus* show an early Th2-dominated immune response (days 7–14), but by day 28 a strongly regulatory profile is evident with antigen-specific IL-10 release and elevated frequency of CD4^+^ T cells bearing surface TGF-β. CD4^+^CD25^+^ T cells from infected mice show enhanced capacity to block *in vitro* effector T cell proliferation. CD4^+^CD25^+^ cell numbers expand dramatically during infection, with parallel growth of both CD25^+^Foxp3^+^ and CD25^+^Foxp3^–^ subsets. CTLA-4 and glucocorticoid-induced tolerance-associated receptor, also associated with regulatory T cell function, become more prominent, due to both expanded CD25^+^ cell numbers and increased expression among the CD25^–^ population. Both intensity and frequency of CD103 expression by CD4^+^ T cells rise significantly, with greatest expansion among CD25^+^Foxp3^+^ cells. While TGF-β expression is observed among both CD25^+^Foxp3^+^ and CD25^+^Foxp3^–^ subsets, it is the latter population which shows higher TGF-β staining following infection. These data demonstrate in a chronic helminth infection that Foxp3^+^ regulatory T cells are stimulated, increasing CD103 expression in particular, but that significant changes occur to other populations including expansion of CD25^+^TGF-β^+^Foxp3^–^ cells, and induction of CTLA-4 on CD25^–^ non-regulatory lymphocytes.

## Introduction

Long-lived helminth parasite infections develop an intriguing balance with the immune system of their host 1, 2. Their continued survival in an immunologically sufficient environment may be ascribed in part to interference with immune activation and attack 3, 4. However, there is increasing evidence that the infected host develops a form of immunological ‘tolerance’ to parasite antigens, which may selectively mute certain effector mechanisms 5. The possibility that susceptibility to helminth infections may be mediated, in part, by regulatory T cells (Treg) is supported by recent work showing that antibody treatment to Treg markers results in heightened anti-parasite responsiveness and clearance of adult worms in *Litosomosoides sigmodontis* infection 6.

Immunological down-modulation during infection is also important in protecting the host from the more pathological outcomes of infection. In the case of schistosomiasis, egg production can cause hepatic granulomatous disease in chronically infected hosts 7. Immunopathology is controlled initially by the regulatory cytokine IL-10, as IL-10-deficient mice succumb to acute liver inflammation 8, while chronic granulomatous fibrosis can be suppressed by T cells transfected with the forkhead box transcription factor p3 (Foxp3) 9, which is functionally associated with Treg activity 10, 11.

The ability of helminth infection to modulate responses to unrelated bystander antigens is well established 12, 13. More recently, it has been recognised that infection can alter the pathological outcome of autoimmune 14, 15 and allergic 16 challenge. Indeed, we recently showed that infection with the murine gastrointestinal nematode *Heligmosomoides* *polygyrus* dampens immune responsiveness to unrelated allergens (ovalbumin and the house dust mite antigen *Der p*1) in a manner dependent upon CD4^+^CD25^+^ T cell activity, but independent of the action of IL-10 17. Moreover, CD4^+^CD25^+^ T cells from the mesenteric lymph nodes (MLN) of *H. polygyrus*-infected, allergen-naïve mice were able to confer suppression of allergy when transferred to uninfected, allergen-sensitized recipients, demonstrating their potent regulatory capacity 17.

Two cardinal characteristics associated with human helminthiases are reproduced in the *H. polygyrus* model of infection. First, this parasite is known to induce a dominant Th2 response 18–23, while secondly it provides an excellent example of generalised down-regulation of immune responsiveness 24–26, attributable in part to the activity of CD4^+^CD25^+^ Treg 17. In the present study, we follow in detail the evolution of both Th2 and Treg parameters over the course of infection, with particular focus on CTLA-4 (CD152), glucocorticoid-induced tolerance-associated receptor (GITR), CD103, TGF-β and Foxp3 within both CD4^+^CD25^+^ and CD4^+^CD25^–^ subsets, as well as the functional characteristics of Treg populations.

## Results

### *H. polygyrus* generates a typical Th2 response early in infection

*H. polygyrus* is a natural gastrointestinal nematode parasite of mice, which follows a direct transmission cycle 27. Orally ingested larvae invade the intestinal mucosa, from where, 9–11 days post-infection, they emerge as adult worms 28. Subsequently, in most strains of mice, they survive as chronic, luminal-dwelling infections that may persist for as long as 300 days 29.

**Figure 1:**
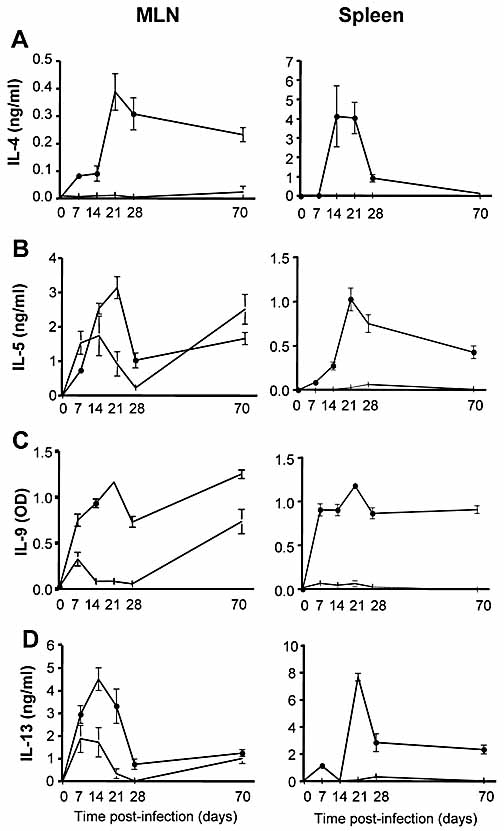
Elevated Th2 cytokine responsiveness in *H. polygyrus* infection. Parasite antigen-specific cytokine responses are presented from MLNC (left) and spleen cells (right) taken at day 7–70 post-infection. Cells were cultured for 48 h in medium alone (open symbols) or *H. polygyrus* antigen (solid symbols). Panels (A–D) present respectively IL-4, IL-5, IL-9 and IL-13. Data represent means ± SE from groups of five mice assayed individually; day 0 represents all naïve mice (five for each time point).

To analyse the adaptive immune response to *H. polygyrus*, we first characterised the cytokine profile of MLN and spleen cells in response to parasite antigen challenge *in vitro*, over the course of a 10-wk infection. As shown in Fig. 1, by day 7 there is a substantial Th2-type response marked by antigen-specific IL-4, IL-5, IL-9 and IL-13 evident in the MLN. Th2 responsiveness remains for the life of the infection although generally down-modulated after day 21. Splenic responses are slower to evolve but follow a similar pattern of Th2 responsiveness, as previously reported 18–23.

**Figure 2:**
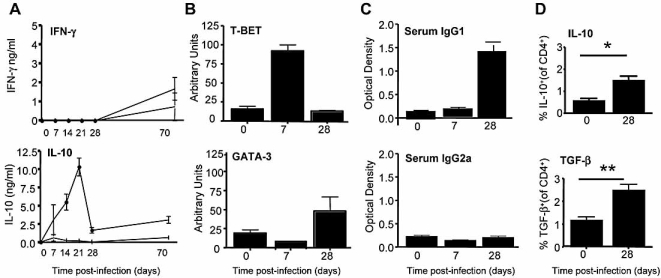
Nascent Th1 and sustained regulatory cytokine responses in *H. polygyrus* infection. (A) IFN-γ and IL-10 release from MLNC directly stimulated *ex vivo*, taken at days 7–70 post-infection and cultured for 48 h in medium alone (open symbols) or *H. polygyrus* antigen (solid symbols). (B) T-BET and GATA-3 real-time PCR in MLN from naïve and infected mice. CD4^+^ T cells were purified from MLN, RNA was extracted and real-time PCR performed on the resulting cDNA. (C) *H. polygyrus*-specific serum antibodies of IgG1 and IgG2a isotypes measured by ELISA. (D) Intracellular IL-10 (left) and surface TGF-β (right) staining in naïve and day 28-infected MLN CD4^+^ T cells determined by flow cytometry. Data represent means ± SE from groups of five mice assayed individually; day 0 represents all naïve mice (five for each time point). *p < 0.05, **p < 0.01.

In contrast, Th1-type responses are relatively feeble. As shown in Fig. 2A, parasite-specific IFN-γ responses *in vitro* are only detectable at day 70, when infection is waning. A nascent Th1 reaction at day 7 can be detected at the mRNA level, evident by raised T-box family transcription factor expressed in T cells (T-BET) expression in RT-PCR (Fig. 2B), at the stage when larval parasites are still resident in the intestinal mucosa. However, by day 28, when adult worms are established in the gut lumen, this T-BET has been replaced by the Th2-promoting factor GATA-3 (Fig. 2B). A further reflection of Th2 polarisation is in the isotype balance of anti-*H. polygyrus* serum antibodies. As previously reported 30, infection stimulates high levels of IgG1, but no detectable IgG2a (Fig. 2C).

### Regulatory cytokine expression

MLN cells (MLNC) from *H. polygyrus* mice respond briskly to parasite antigen challenge *in vitro* with IL-10 release over the course of infection (Fig. 2B). Moreover, *ex vivo* staining of MLNC from day 28-infected mice shows significant increases in both intracellular IL-10 and surface-bound TGF-β (Fig. 2D). These findings are consistent with other reports of elevated antigen-specific and serum TGF-β in mice infected with *H. polygyrus* 31.

### Expansion of CD25^+^ T cells during infection

**Figure 3:**
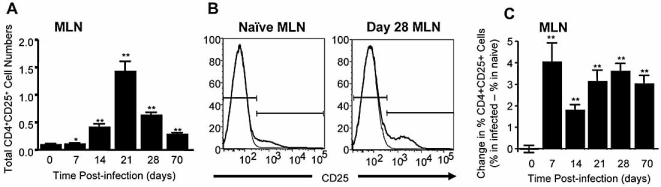
Expansion of CD25-expressing cells within the total CD4^+^ T cell population during *H. polygyrus* infection. (A) Total numbers of CD4^+^CD25^+^ T cells from day 7–70 post-infection in MLN. (B) Representative histograms of CD25 expression within MLN (left) or splenic (right) CD4^+^ cells from day 28-infected (black) or naïve (grey) mice; isotype controls are shown as thick black lines. (C) Changes in proportion of CD25-expressing CD4^+^ T cells relative to total CD4^+^ cells over 70 days of infection. For each point in time, the percentages of CD4^+^ T cells which also expressed CD25 among MLNC from infected and naïve animals were determined; the arithmetic difference between infected and naïve in percentage frequency at each time point was then calculated. The mean naïve level was 14.36% (SD = 0.80). For (A, C), Mann–Whitney tests were performed (n.s., no significant difference; *p<0.05, **p<0.01). Data represent means ± SE from groups of five mice assayed individually, compared with naïve mice at the same assay time. Day 0 represents all naïve mice (five for each time point).

**Figure 4:**
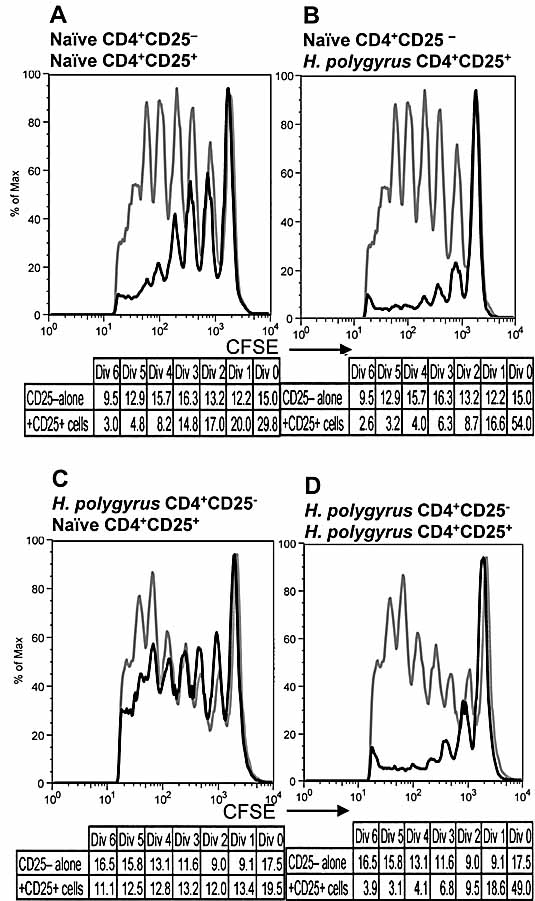
CD4^+^CD25^+^ T cells from *H. polygyrus*-infected mice show more potent suppression of CD25^–^ effector cell proliferation. Proliferation of 5×10^4^ CD4^+^CD25^–^ T cells in response to *in vitro* Con A stimulation was measured by CFSE (grey lines), in populations from naïve (A, B) and day 28-infected (C, D) MLNC. In parallel cultures, equal numbers of CD4^+^CD25^+^ MLNC were added to the CD4^+^CD25^–^ CFSE-loaded T cells. MLNC from infected mice (B, D; black lines) show more profound suppression than equal numbers of cells from uninfected controls (A, C; black lines). Tables beneath each graph give percentage of CD25^–^ cells in each round of division when stimulated in the absence (upper row) or presence (lower row) of CD25^+^ cells. Data represent pooled cells from groups of five mice.

Expression of the IL-2Rα chain, CD25, is a widely used but not exclusive marker for Treg 32. Using flow cytometry, we found that total CD4^+^CD25^+^ cell numbers expand dramatically in the draining MLN (Fig. 3A), although total LN cell counts are expanding during the same period. A smaller rise in CD25^+^ cells occurs in the spleen, particularly from day 21 onwards (data not shown). The intensity of CD25 expression on positive cells does not alter appreciably (Fig. 3B). When considered as a proportion, it can be seen that CD4^+^CD25^+^ cells outgrow CD4^+^CD25^–^ cells from day 7 onwards and remain in significant excess over uninfected controls throughout infection (Fig. 4C).

### *In vitro* suppressive activity by CD25^+^ Treg

A common test of functional Treg activity is their ability to block the proliferative response of effector T cells to antigen or mitogen stimulation 33. We accordingly assessed whether sorted CD4^+^CD25^+^ MLNC from infected animals were able to suppress the proliferation of CFSE-loaded naïve, CD4^+^CD25^–^ MLNC, responding to Con A. We observed that, on a per-cell basis, CD4^+^CD25^+^ MLNC, taken 28 day post-infection, were substantially more suppressive than cells with similar phenotype from naïve animals (Fig. 4A, B).

In parallel experiments we also noted that CD4^+^CD25^–^ T cells from infected mice showed greater resistance to proliferative inhibition, and indeed were largely refractory to suppression by CD4^+^CD25^+^ MLNC from naïve mice (Fig. 4C). However, CD4^+^CD25^–^ effectors from *H. polygyrus*-infected animals remained susceptible to inhibition by the more ‘activated’ regulatory cells from infected mice (Fig. 4D, E).

### Foxp3 expression levels remain relatively constant during infection

**Figure 5:**
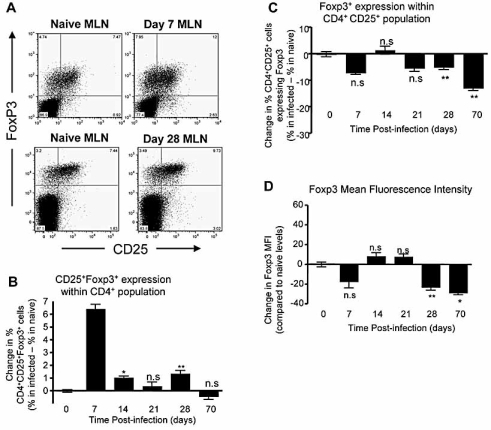
CD4^+^ T cell Foxp3 expression levels remain relatively constant during *H. polygyrus* infection. (A) Bivariate flow cytometry analysis of CD25 and Foxp3 expression in CD4^+^ T cells in naïve (far left), day 7-infected (left) and naïve (right) and day 28-infected (far right) MLNC. (B) Time course of expression of CD25^+^Foxp3^+^ as percentage of total CD4^+^ cells in MLN from 7–70 days of infection. The mean naïve level was 6.43% (SD = 2.27). (C) Foxp3^+^ T cells as proportion of total CD4^+^CD25^+^ T cells in naïve and day 28-infected MLN. The mean naïve level was 67.2% (SD = 17.1). (D) Foxp3 expression intensity over the course of infection. The percentage difference in MFI relative to uninfected values for Foxp3 was calculated. For (B–D) data represent means ± SE from groups of five mice assayed individually; day 0 represents all naïve mice (five for each time point). Mann–Whitney tests were performed (n.s., no significant difference; *p<0.05, **p<0.01).

A key marker for natural Treg is the transcription factor Foxp3, which is expressed by the majority of CD25^+^ T cells 10, 11, 34. As previously reported 17, we found that *H. polygyrus* infection results in a modest increase in CD4^+^CD25^+^Foxp3^+^ cells (from 7.4% of all CD4^+^ cells at day 0 to 9.7% at day 28; Fig. 5A). Expansion occurs rapidly following infection (Fig. 5B), although, due to parallel expansion of CD25^+^Foxp3^–^ cells in infection, there is in fact a small diminution in the proportion of CD4^+^CD25^+^ cells which express Foxp3 (Fig. 5C). It was also apparent that the intensity of Foxp3 expression within the Foxp3^+^CD4^+^CD25^+^ T cell population does not increase (Fig. 5D). Thus, the increment in CD25^+^ cells during infection is not accompanied by preferential Foxp3 induction and represents an expansion of both CD4^+^CD25^+^Foxp3^+^ and CD4^+^CD25^+^Foxp3^–^ T cells. This latter phenotype may represent either an activated effector cell (which could be stimulated in the environment of chronic infection), or a Foxp3^–^ ‘adaptive’ or inducible Treg.

### Increased frequency of CTLA-4 and GITR expression during infection

**Figure 6:**
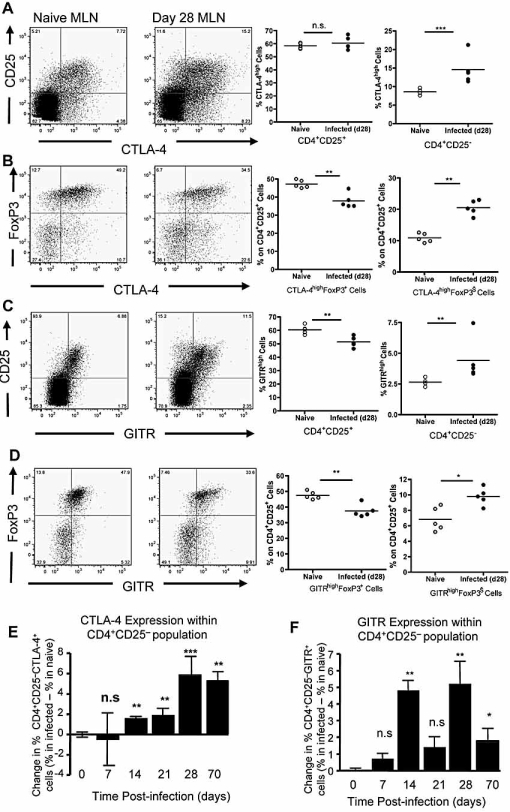
CTLA-4 and GITR expression levels increase on CD4^+^CD25^–^ cells, but not on CD4^+^CD25^+^ cells, during *H. polygyrus* infection. (A) CTLA-4 expression measured by flow cytometry on MLNC. (Left panels) CTLA-4 and CD25 bivariate plot from naïve and day 28-infected mice; (right panels) percentage of CTLA-4^high^ T cells in the CD4^+^CD25^+^ and CD4^+^CD25^–^ subsets. (B) CTLA-4 expression measured by flow cytometry on MLNC. (Left panels) CTLA-4 and Foxp3 bivariate plot from naïve and day 28-infected mice; (right panels) percentage of CTLA-4^high^ T cells in the CD25^+^Foxp3^+^ and CD25^+^Foxp3^–^ subsets. (C) GITR expression, measured as for CTLA-4 in Fig. 7A. (D) GITR expression, measured as for CTLA-4 in Fig. 7B. (E) Frequency of CTLA-4 expression in CD4^+^CD25^–^ cells over the course of infection. Mean naïve level = 6.58% (SD = 1.82). (F) Frequency of GITR expression in CD4^+^CD25^–^ cells over the course of infection. Mean naïve level = 5.45% (SD = 4.75). Data represent means ± SE from groups of five mice assayed individually. Mann–Whitney tests were performed (n.s., no significant difference; *p<0.05, **p<0.01, ***p<0.001.

Two surface markers closely associated with the Treg phenotype are CTLA-4 35 and GITR 36. Both were measured in MLN and spleen cells over the 70-day time course. At day 28, for example, CTLA-4 is expressed by 23% of CD4^+^ T cells, compared to 12% in naïve controls (Fig. 6A). This increase is represented by a relatively constant per-cell level within the CD4^+^CD25^+^ population, together with a significant rise in expression among CD4^+^CD25^–^ cells (Fig. 6A). Moreover, within the CD4^+^CD25^+^ compartment (Fig. 6B) CTLA-4 staining increases more than twofold among the Foxp3^–^ subset, while actually declining in Foxp3^+^ cells. A similar, though less marked, trend is seen with GITR staining; overall GITR expression within the CD4^+^ T cell population rises from 8.5% in naïves to 13.8% in day 28-infected mice (Fig. 6C). Again there is a significant rise in the proportion of CD4^+^CD25^–^ cells expressing GITR, and within the CD4^+^CD25^+^ subset the expansion in GITR^+^ cells occurs with the Foxp3^–^ population (Fig. 6D). These changes are sustained over the longer term course of infection (Fig. 6E, F). Thus, the uplift in CTLA-4 and GITR is observed primarily, if not totally, within cells of a non-regulatory phenotype.

### CD103 and TGF-β expression is raised in frequency and intensity by infection

**Figure 7:**
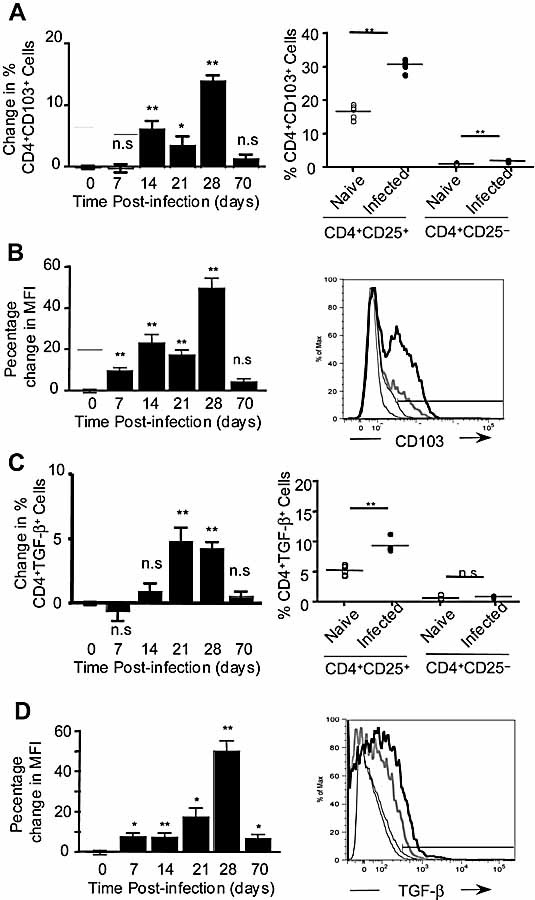
CD103 and TGF-β expression is raised in frequency and intensity by *H. polygyrus* infection. MLNC were stained for CD4, CD25, CD103 and TGF-β and analysed by flow cytometry. For CD103 and TGF-β, the percentages within total CD4^+^CD25^+^ T cell populations were calculated, as well as the percentage change in MFI for each infected group compared to the corresponding naïve group; Mann–Whitney tests were performed (n.s., no significant difference; *p<0.05, **p<0.01). Data represent means ± SE from groups of five mice assayed individually. Day 0 represents all naïve mice (five for each time point). (A) CD103^+^ T cell numbers over the time course (left), and as proportion of total CD4^+^CD25^+^ or CD4^+^CD25^–^ T cells in naïve and day 28-infected MLNC (right). Over the time course, mean naïve level = 18.3% (SD = 4.09). (B) CD103 expression levels over the time course (left) and in representative MLNC from naïve (grey line) and day 28-infected (thick black line) mice (right); isotype controls are shown as thin black lines. (C) TGF-β^+^ cell numbers over the time course (left), and as proportion of total CD4^+^CD25^+^ or CD4^+^CD25^–^ T cells in naïve and day 28-infected MLNC (right). Over the time course, mean naïve level = 8.01% (SD = 2.67). (D) TGF-β expression levels over the time course (left), and in representative MLNC from naïve (grey line) and day 28-infected (thick black line) mice (right); isotype controls are shown as thin black lines.

We also examined expression of CD103 (the integrin αEβ7) and of the regulatory cytokine TGF-β in the CD4^+^ populations. In the MLN, there were significant increases in the proportion of CD4^+^CD25^+^ T cells which express CD103, returning to control levels by day 70 of infection (Fig. 7A); a similar pattern was observed in splenic populations (data not shown). While most CD103-expressing cells were in the CD4^+^CD25^+^ subset, there were also small but significant increases in the frequency of CD103^+^ among CD25^–^ cells. Moreover, infection induced a substantial upshift in intensity of CD103 expression, reaching levels approximately 50% higher than in naïve populations (Fig. 7B).

A similar profile was observed for surface-bound TGF-β. A modest, but significant, rise in TGF-β staining occurred in both MLN and splenocytes, peaking at day 21–28; thus by day 28 nearly 10% of all CD4^+^CD25^+^ T cells were TGF-β^+^, although no change was seen in the very low levels of TGF-β among CD4^+^CD25^–^ cells (Fig. 7C). There was also a measurable rise in fluorescence intensity, evident only in the CD4^+^CD25^+^ population, over the first 28 days of infection (Fig. 7D).

### CD25^+^Foxp3^+^ cells express higher CD103, and CD25^+^Foxp3^–^ cells show raised TGF-β expression

**Figure 8:**
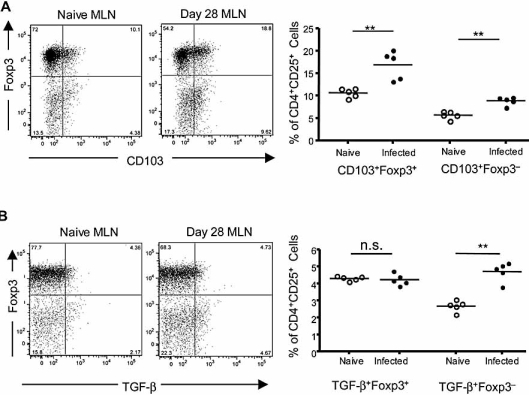
By day 28 of infection, CD103^+^Foxp3^+^ cells increase whilst TGFβ^+^Foxp3^+^ do not. Cells were stained for CD4, CD25, CD103, Foxp3 and TGF-β. Levels of CD103, TGF-β and Foxp3 expression were determined within the CD4^+^CD25^+^ population. Mann–Whitney tests were performed (n.s., no significant difference; **p<0.01). (A) CD103 expression plotted by bivariate analysis against Foxp3 staining, in naïve and day 28-infected MLNC (left), and percentage expression of CD103^+^Foxp3^+^ and CD103^+^Foxp3^–^ cells within CD25^+^ T cell populations (right). (B) Surface TGF-β expression plotted by bivariate analysis against Foxp3 staining, in naïve and day 28-infected MLNC (left), and percentage expression of TGF-β^+^Foxp3^+^ and TGF-β^+^Foxp3^–^ cells within CD25^+^ T cell populations (right).

Since the CD4^+^CD25^+^ population from infected mice displayed increases in both CD103 and TGF-β, co-staining was performed for these markers and for the Foxp3 transcription factor. These analyses showed, firstly, that in naïve MLNC approximately two-thirds of the CD103^+^ cells are Foxp3^+^ (Fig. 8A), as are a similar proportion of the TGF-β^+^ cells (Fig. 8B). However, infection generated a substantial and significant increase in CD25^+^CD103^+^Foxp3^+^ cells (Fig. 8A), while the frequency of TGF-β^+^Foxp3^+^ cells did not differ between naïve and infected mice (Fig. 8B). Moreover, we found that both CD103 and TGF-β staining occurs on a significant subset of CD25^+^Foxp3^–^ cells, and indeed comparison of naïve and infected MLN shows significant increases in CD103^+^Foxp3^–^ (Fig. 8A) and in TGF-β^+^Foxp3^–^ cells (Fig. 8B).

## Discussion

It is now evident that Treg are active at many points in the control of immune responses against pathogens 37–41. CD4^+^CD25^+^ Treg block protective immunity in animal models of malaria 42 and filariasis 6, but are also required to minimise pathology caused by the response to pathogen invasion 43–45. This critical balance between benefit and harm is played out against a backdrop of pathogens which are likely to have evolved strategies to favour Treg priming, recruitment and survival 41. For the host, the optimal regulatory response may permit residual parasite survival, so providing ongoing antigenic stimulation without disease 38. Thus, whether considering susceptibility, pathology or immunity to pathogens, the contribution of Treg populations can prove decisive.

Helminth parasitic diseases are typically slowly evolving and chronic in nature, often associated with immune down-regulation 1, 5. Evidence from human lymphatic filariasis, onchocerciasis and schistosomiasis records a down-modulation of immunity which is consistent with the activity of Treg-like cells, involving IL-10, TGF-β and CTLA-4 1, 46–49. In animal models of filariasis 50 and schistosomiasis 51, 52, Treg phenotype populations develop following infection, whilst in infection with the murine gastrointestinal nematode *H. polygyrus* 17, functional regulation by CD4^+^CD25^+^ T cells suppresses the bystander response to an allergic provocation. In the current study, we also show that this phenotype is enhanced, following infection, in its ability to suppress the polyclonal proliferative response of CD25^–^ effector cells. However, the origin and specificity of the CD4^+^C25^+^ population generated by this or other helminth infections have yet to be delineated.

CD25 (IL-2Rα) is not a unique marker for Treg, being also expressed on activated effector T cells 33. For this reason we analysed additional functional and phenotypic markers, including the transcription factor Foxp3 10 and the inhibitory/stimulatory co-receptors CTLA-4 35 and GITR 36, 53. Expression of Foxp3, as well as the production of the suppressive cytokines IL-10 and TGF-β, are considered to distinguish natural or adaptive Treg subsets 54.

Broadly, natural Treg arise during the normal process of maturation in the thymus, are selected on the basis of specificity for self antigens, express surface CD25 and Foxp3, and employ cell contact-dependent suppressive mechanisms. In contrast, adaptive Treg are thought to develop from naïve (CD25^–^, Th0) mature peripheral populations in response to specific stimulatory conditions such as sub-optimal signalling from accessory cells. Adaptive Treg include those designated Tr1 55 and Th3 56, produce IL-10 or TGF-β, and have induced CD25 expression. Whether these cells also initiate Foxp3 expression is controversial. No induction could be found either in Tr1 induced *in vitro* with IL-10, or *in vivo* by intranasal tolerisation 57. However, *in vitro* CD4^+^CD25^–^ T cells can be induced to express CD25 and Foxp3 following stimulation with TGF-β 58–60, and in a T cell receptor-transgenic, thymectomised mouse, *de novo* Foxp3 expression was induced in a regulatory CD25^+^ population exposed to low-dose antigen delivered by osmotic pump 61. Hence, Foxp3 expression delimits a subset containing all naturally arising Treg and, possibly, a proportion of adaptive Treg.

In *H. polygyrus* infection, there is a preferential expansion of CD25-expressing cells without a proportional increase in Foxp3^+^ cells, and yet the functional regulatory activity of the CD25^+^ T cell population is greatly amplified in infected mice. Hence, the increment in CD25^+^Foxp3^–^ T cell numbers is unlikely to represent effector cell expansion alone. A plausible hypothesis is that many of the CD25^+^Foxp3^–^ cells are adaptive Treg, with specificity for parasite antigens, which have arisen from naïve precursors with induction of expression of CD25 rather than that of Foxp3. In addition, there is good evidence from our work and that of others, that production of IL-10 and TGF-β is substantially heightened in *H. polygyrus* infection 17, 31.

Treg-derived IL-10 is a major determinant in systems where Th1 immune responses are protective, such as murine infection with *Leishmania major* 38. In schistosome infections which drive dominant type-2 responsiveness, however, only a small proportion of the IL-10 emanates from CD25^+^Foxp3^+^ T cells 51, 52, and even though IL-10 is important in the overall control of immune pathology in schistosomiasis, granuloma modulation is IL-10-independent 8. Moreover, IL-10 is a critical promoter of strong Th2 responses in many helminth systems 62, and unlike the Th1 setting does not fulfil a purely down-regulatory role. This is confirmed in the case of *H. polygyrus*, as the ability of CD25^+^ Treg to suppress airway allergy in infected mice is undiminished by anti-IL-10R antibody, while MLNC from IL-10-deficient infected mice transfer suppression of allergy into uninfected animals 17. Hence, in the mouse at least, IL-10 does not appear to be a primary mechanism for helminth-associated Treg function.

TGF-β, however, remains a credible candidate for the functional Treg product in this system, with respect to both the induction and survival of Treg 63, and the down-modulation of effector T cell populations 64. We found significantly raised surface TGF-β staining over the course of infection, and others have reported parasite antigen-specific TGF-β release in similar experiments 31. Most recently, Doligalska and colleagues 65 have reported that anti-TGF-β antibody treatment greatly reduces egg production and worm survival in mice, indicating an important role for TGF-β in parasite immune evasion. Interestingly, in our experiments, increased TGF-β was observed within the CD25^+^Foxp3^–^ subset, and not among CD25^+^Foxp3^+^ cells. Hence, the cells induced to express TGF-β may be adaptive Treg most similar to the Tr1/Th3 type described in other systems, in particular the TGF-β-secreting cells derived from the MLN of orally tolerised mice 56.

We also observed a marked increase in CTLA-4 expression among T cells from infected mice. CTLA-4 is an inhibitory component of the co-stimulation machinery associated with T cell receptor signalling, and may act by competing for ligand with the CD28 stimulatory receptor, and by raising the activation threshold of T cells through the immunological synapse 66. Hence, when CTLA-4 is blocked by antibody treatment *in vivo*, parasite expulsion is accelerated 67. Interestingly, the more substantial upshift in CTLA-4 staining was seen on CD25^–^ cells, and this change occurred later in the course of infection than the expansion in CD25^+^ cell numbers. It is plausible, therefore, that the CD25^–^CTLA-4^+^ phenotype represents an anergic effector cell, as postulated in both human 48, 49 and mouse 6 helminth infections, which develops subsequent to and under the influence of the more rapidly arising Treg response to infection.

GITR, originally identified as a marker up-regulated on Treg, is a receptor thought to activate both regulatory and effector cells on ligation. In *H. polygyrus* infection, levels of GITR expression increase albeit less than observed for CTLA-4. In particular, CD25^–^ T cells as well as Foxp3^–^CD25^+^ cells show small but significant uplifts in the frequency of GITR expression. The induction of GITR on the CD25^–^ population is similar to that observed in the tissue helminth infection, *L. sigmodontis* 6.

Treg may act either, or both, at the induction stages of the immune response and at the inflammatory phase in the tissues. In *H. polygyrus* infections, priming to allergens is unaffected and infection-generated Treg transferred into fully primed mice suppress inflammation in the lung 17. If infection-induced Treg act primarily at the site of inflammation, this may be reflected in the pattern of homing marker expression 68, 69. In this context, the marked up-regulation of CD103 on the infected CD4^+^CD25^+^ T cell subset may have particular functional significance. CD103 is a homing marker and is expressed on 20–30% of Treg in lymphoid organs 53. CD103, therefore, may not be a mechanistic mediator of suppression, but rather a pre-requisite for Treg to traffic into, and remain at, sites of inflammation. In a model of leishmaniasis, CD103 is induced and maintained on Treg following or just prior to their arrival in inflamed tissues 70. Hence, CD103 does not define a lineage of CD25^+^ Treg with distinct properties, but rather a subset capable of homing into the site of infection. The expression of CD103 is positively regulated by TGF-β 71. Surface TGF-β levels increase during infection, and this may provide a mechanism by which CD103 is continuously up-regulated.

In conclusion, we show here that *H. polygyrus* induces significant phenotypic changes in distinct subsets of cells, including one with functional suppressive properties and some characteristic markers associated with Treg. It may be significant that the Foxp3-expressing population is only one of the players in the system, and we cannot yet distinguish whether ‘natural’ and ‘adaptive’ Treg expand independently in the context of this infection, or the evolution of the adaptive phenotype is dependent upon the pre-existing natural Treg population. Further characterisation of these regulatory cells, their antigen specificity and their mechanisms is therefore imperative, as is the analysis of the potentially anergic effector T cell population which we have postulated. Nematode infections are of particular importance since they affect over two billion people worldwide, mostly in poverty-stricken regions where numerous other infections are rife. Unravelling the effects of dampened immune responses, due to increased regulatory mechanisms triggered by worms, on disease progression and outcome in co-infected people would open new avenues for treatment, control and eradication of these prevalent diseases.

## Materials and methods

### Mice and parasites

Female BALB/c mice, 6–8 wk of age and maintained in individually ventilated cages, were infected with 200 *H. polygyrus bakeri* infective L3 larvae using a gavage tube. In time-course experiments, a matched group of naïve animals was analysed at each time point taken.

### *H. polygyrus* antigen and ELISA

*H. polygyrus* antigen was prepared by homogenising adult worms in PBS, which was centrifuged (13 000 × *g*, 10 min); the supernatant was filtered (0.2 µm Millex) and stored at 1.5 mg/mL at –80°C. Antigen-specific antibody responses were determined by ELISA. Multisorp (Nunc) plates were coated with 5 μg/mL *H. polygyrus* antigen in 0.06 M carbonate buffer pH 9.6, overnight at 4°C. Plates were blocked with 5% BSA (fraction V, Gibco) for 2 h at 37°C. Sera were diluted in TBS/0.05% Tween and added to wells overnight at 4°C. Antigen-specific IgG isotypes were detected with HRP-conjugated goat anti-mouse IgG1 (1070-05, Southern Biotechnology) and anti-IgG2a (1080-05), with ABTS peroxidase substrate (50-62-00, KPL).

### *In vitro* restimulation and cytokine assays

Unfractionated LN and spleen cells were cultured at 1×10^7^/mL in 96-well plates (3799, Costar) for 48 h in RPMI 1640 medium (Gibco), 10% FCS, 1% l-glutamine, 1% penicillin/streptomycin (supplemented RPMI 1640) in the presence of 10 μg/mL *H. polygyrus* antigen or 1 μg/mL Con A (Sigma). Cytokines in supernatants were measured by ELISA according to suppliers’ guidelines. Capture antibodies for IL-4 (11B11, 4 μg/mL), IL-5 (TRFK5, 2 μg/mL), IL-10 (JES5-2A5, 4 μg/mL) and IFN-γ (R46A2, 3 μg/mL) were produced in-house or by Pharmingen. Capture antibody for IL-13 (38213, 2 μg/mL) was from R&D Systems, and that for IL-9 (229.4, 5 µg/mL) kindly provided by Dr. Melanie Leech (IIIR, Edinburgh). Biotinylated detection antibodies were from Pharmingen: IL-4 (BVD6-24G2, 5 μg/mL), IL-5 (TRFK4, 2 μg/mL), IL-9 (D9302C12, 1 μg/mL), IL-10 (SXC-1, 2 μg/mL) and IFN-γ (XMG1.2, 0.5 μg/mL), or for IL-13, from Peprotech (rabbit polyclonal, cat. No. 500-P178Bt, 0.1 μg/mL).

### Flow cytometry

LN and spleen cell suspensions were prepared for flow cytometry at 1×10^7^/mL in supplemented RPMI 1640. Antibodies were diluted in PBS, 0.5% BSA (Sigma), 0.05% sodium azide, and added to cell suspensions (1×10^6^–2×10^6^ total cells) for 20 min at 4°C. For detection of CD4^+^CD25^+^ and CD4^+^CD25^–^ cells, rat anti-mouse CD4 (L3T4, clone RM4-5, IgG2a, 1/100) and anti-CD25 (clone PC61, IgG1, CALTAG, 1/100) monoclonal antibodies were used. For intracellular IL-10 and CTLA-4 staining, cells were permeablised in cytofix/cytoperm, washed in perm/wash buffer (Pharmingen) and stained with rat anti-mouse IL-10 (JES5-16E3, rat IgG2b, 1/50) or CTLA-4 (UC10-4F10-11, 1/10) for 30 min. For staining Foxp3, cells were permeablised in cytofix/cytoperm for 1 h, washed in perm/wash buffer (eBiosciences) and stained with rat anti-mouse Foxp3 (FJK-16s, rat IgG2a, eBiosciences, 1/50) for 30 min. Surface-bound TGF-β, CD103 and GITR were detected using rat anti-mouse TGF-β1 (A75-3, IgG2a, 1/25), CD103 (M290, IgG2a, 1/100) and GITR (DTA-1, IgG2a, produced in-house, 1/250). Analysis was performed on an LSR II flow cytometer using FlowJo software (Tree Star). All fluorochrome-labeled antibodies were obtained from BD Pharmingen, unless otherwise stated.

### CD4^+^ T cell enrichment and quantitative PCR

For CD4^+^ cell purification, cell suspensions were incubated at 1×10^8^/mL with 10 μL CD4 (L3T4) microbeads (130-049-201, Miltenyi Biotech) per 10^7^ cells and separated on MACS MS columns with pre-separation filters. RNA was recovered from purified CD4^+^ cells by the addition of Trizol (Invitrogen, 1 mL/10^6^ cells), and extracted following the manufacturer's protocol. RT-PCR was performed with 1 μg RNA using murine Moloney leukaemia virus reverse transcriptase (Stratagene) and oligo(dT) primers (Promega). Transcript quantification was performed by real-time RT-PCR, using the LightCycler (Roche Molecular Biochemicals), with *β-actin* for normalisation.

PCR amplifications were in 10 μL, containing 1 μL cDNA, 1.2 μL MgCl_2_ (25 mM), 0.3 μL (10 μM) primers and 1 μL LightCycler-DNA SYBRGreen-I mix (10×). For *Foxp3* amplification the QuantiTech SYBRGreen PCR Kit (Qiagen) was used, and PCR amplifications were performed in 10 μL, containing 1 μL cDNA, 0.5 μL (10 μM) primers and 5 μL SYBRGreen mix. The amplification of *β-actin* (5′-TGGAATCCTGTGGCATCCATGAAAC-3′, 5′-TAAAACGCAGCTCAGTAACAGTCCG-3′), *T-bet* (5′-GCCAGGGAACCGCTTATATG-3′, 5′-GACGATCATCTGGGTCACATTGT-3′)*, Smad-7* (5′-GCATTCCTCGGAAGTCAAGAGG-3′, 5′-TGCGGTTGTAAACCCACACG-3′)*, Foxp3* (5′-CCTGGCCTGCCACCTGGGATCAA-3′, 5′-TTCTCACAACCAGGCCACTTG-3′) and *Gata-3* (5′-CTACGGTGCAGAGGTATCC-3′, 5′-GATGGACGTCTTGGAGAAGG-3′) was performed as follows: 30 s denaturation at 95°C, 5 s annealing at 55°C and 12 s elongation at 72°C, for 40–60 cycles. For *T-bet* the acquisition temperature was reduced to 84°C. Conventional curve analyses were performed according to the LightCycler instruction kit. Products were run on agarose to ensure that no genomic DNA amplification occurred.

### CD25 enrichment and suppression assay

For CD4^+^CD25^+^ cell enrichment, CD4^+^ cells were negatively isolated using sheep anti-rat IgG beads (M540, Dynal) and biotinylated anti-MAC1 (0.5 μL/10^7^ cells, M1/70.15), anti-CD8α (0.5 μL/10^7^ cells, 53-6.72), anti-MHC class II (1 μL/10^7^ cells, M5114), anti-B220 (0.5 μL/10^7^ cells, RAB632) and anti-GR1 (3.33 μL/10^7^ cells, RB6-8C5, BD Pharmingen). Antibody-bound beads and cell solutions were separated on a magnetic particle concentrator (Dynal MPC). Positive selection of CD25^+^ cells then employed PE-conjugated anti-CD25 (130-091-013, Miltenyi Biotech) and PE microbeads (130-048-801, Miltenyi Biotech), on MACS LS separation columns with pre-separation filters. CD25^–^ cells obtained were stained with CFSE (5 μM, Sigma) and cultured with or without 5×10^4^ CD4^+^CD25^+^ cells for 4 days in a 1:1 ratio, with 1×10^5^ irradiated CD4^–^ APC and in the presence of 1 μg/mL Con A.

### Statistics

Mann–Whitney test was used for all statistical comparisons; *p* values less than 0.05 were considered significant. To compare the percentage of cells positive for the surface and intracellular markers studied, the difference in frequency of the marker was calculated between each infected group and its naïve group. For comparing intensity levels, the difference in each infected group compared to its naïve group was calculated as a percentage increase/decrease compared to the baseline naïve values.

## Abbreviations:

Foxp3: forkhead box transcription factor p3
GITR: glucocorticoid-induced tolerance-associated receptor
MLNC: MLN cells
T-BET: T-box family transcription factor expressed in T cells
